# Correction: Effects of students’ mathematics learning strategy and perceived task difficulty on their achievement of mathematics

**DOI:** 10.1371/journal.pone.0355797

**Published:** 2026-08-11

**Authors:** Woldeab Daniel Eka, Tiruwork Tamiru Tola, Reda Darge Negasi

## Notice of Republication

This article [1] was republished on July 22, 2026, to address errors in the author affiliations, and to update the ethics statement with the specific name of the approving body. Please download this article again to view the correct version. The originally published, uncorrected article and the republished, corrected article are provided here for reference.

## Supporting information

S1 File Originally published, uncorrected article. (PDF)

S2 File Republished, corrected article. (PDF)
