# Supplementary material for: Correction: Effects of students’ mathematics learning strategy and perceived task difficulty on their achievement of mathematics
Source: PLoS One. 2026 Aug 11;21(8):e0355797. doi: 10.1371/journal.pone.0355797 (PMC13460563; doi:10.1371/journal.pone.0355797)
Supplement: Supplementary file 1 [file pone.0355797.s001.pdf]

PLOS One

RESEARCH ARTICLE

Effects of students' mathematics learning strategy and perceived task difficulty on their achievement of mathematics

Woldeab Daniel Eka[1*], **Tiruwork Tamiru Tola**[2☯], **Reda Darge Negasi**[2☯]

1 Department of Psychology, Wolaita Sodo University, College of Educational and Behavioral Sciences, Wolaita Sodo, Ethiopia, **2** Department of Psychology, Bahir Dar University, College of Educational and Behavioral Sciences, Bahir Dar, Ethiopia

☯ These authors equally contributed to this work.
* woldeab.daniel@wsu.edu.et, woldaniel@yahoo.com

Check for updates

OPEN ACCESS

Citation: Eka WD, Tola TT, Negasi RD (2025) Effects of students' mathematics learning strategy and perceived task difficulty on their achievement of mathematics. PLoS One 20(11): e0337115. https://doi.org/10.1371/journal.pone.0337115

Editor: Suherman Suherman, Universitas Islam Negeri Raden Intan Lampung, HUNGARY

Received: June 21, 2025

Accepted: November 4, 2025

Published: November 21, 2025

Copyright: © 2025 Eka et al. This is an open access article distributed under the terms of the Creative Commons Attribution License, which permits unrestricted use, distribution, and reproduction in any medium, provided the original author and source are credited.

Data availability statement: Data are available on request. This is possible through contacting the chairperson of Institutional Research Review Committee (IRRC), namely, Jerusalem Yibletal Yizengaw as "jeryinternational@gmail.com" through which the request of the underlying data for this study can be addressed.

Abstract

This study examined effects of Mathematics learning strategy and perceived-task-difficulty on their achievement. Post-positivism paradigm, quantitative approach and correlations design were employed. Out of 2893 total student population, 351 were sampled using systematic random sampling. Whilst learning strategy and perceived-difficulty were measured using questionnaire, Mathematics achievement was measured teacher-made tests. Pilot study was conducted on 140 samples and the result revealed that Cronbach alpha for both scales appeared above 0.7. The structural equation modeling (SEM) was employed to analyze the data. The path analysis indicated that 22% of variance in mathematics achievement was significantly explained by the joint effects of learning strategy and perceived task difficulty. The result also indicated that the standardized regression weights of ($\beta = .186$) and ($\beta = -.374$) for learning-strategy and perceived task-difficulty, respectively, were found statistically significant. In conclusion, the effect of Mathematics learning strategy and perceived task difficulty on students' Mathematics achievement is significant, and positive actions on improving task difficulty and Mathematics learning strategy can enhance Math achievement. Hence, setting tasks with proper level of difficulty and assuring students' implementation of learning strategy suitable to contents are essential for enhancing students' achievement in Mathematics.

1 Introduction

1.1 Background and justification

At secondary school level in Ethiopia, Mathematics education is expected to the attainment of essential mathematical knowledge and skill which contribute to creating citizens who are abundantly aware of the socio-economic arena of their country [1]. Due to this, learning Mathematics by anticipating its multifaceted application on daily

Funding: The author(s) received no specific funding for this work.

Competing interests: The authors have declared that no competing interests exist.

routines is unequivocally crucial. This is not merely because Mathematical skills and knowledge are vital in our day to day life [2], but also giving less emphasis to these skills denies students' opportunities to learn numerous other subjects which makes barrier on their job opportunities, and potentially, pool society can be created [3].

Students' attainment of learning outcomes in subjects across grade levels is manifested by their achievement in it [4], and like other subjects, achievement in Mathematics is a function of several factors interplay. Studies indicate that students' learning strategies [5–7], their perception of the subject [8] and strongly held labeling of Mathematics as a difficult subject by students [9], among others, as factors associated with students' achievement.

It is stated by [10] that boosting confidence to deal with Mathematical problems and perseverance on doing, which are associated with selection and implementation of suitable learning strategy, are essential for students to thrive. This indicates us that a strategy that can help dealing with new mathematical contents may not suit properly for enhancing problem-solving or critical thinking skills and vice versa. Due to this, a learner is expected to be familiar with different learning strategies which can be properly applied to different contents.

As stated by [11] that perceived task difficulty is a students' subjective evaluation of tasks as difficult to go through, which differ from self-efficacy beliefs or competence perceptions. Students' perception of Math task difficultly is also expected to have role on their achievement in it. Task difficulty, both the perceived and actual, can predict students' academic achievement. In line with this conviction, [12] stated that students who have high perceived task difficulty may not be motivated to persist at the task. However, still students' perception of task difficulty can also be seen from another angle. For instance, if teacher sets too easy tasks for students, the desire to engage in the task can be reduced as a student perceives that the goal can be attained effortlessly or with very minimum efforts, hence, will devote insufficient time for the task.

It is increasingly becoming common news that performance of students in Mathematics has been worry to sectors in education [13]. More specifically, in context of Ethiopia, low students' result in grade 12 national exam registered in the three consecutive academic years from 2021/22 and 2023/24 [14] has pointed that there is gap to be filled by both teachers and students to change the figure. Actually, it is worrisome that students' achievement in many subjects is decreasing [15], and with regard to Mathematics, several reforms undertaken to alleviate the problem were not effective [16].

Though there have been studies aimed at unveiling why students achieve low in Mathematics, they are not adequate. There have been periodic national reports on students' achievement like National learning assessments (NLA), as stated by [16,17] those assessments are general reports and failed to show the amount of variance in achievement predicted by the student-related variables like perceived task difficulty and Mathematics learning strategies.

There were studies on the effects of students' Math learning strategies on their achievement with identified gaps which have necessitated the current study. Author [18] has investigated how learning strategies affect students' performance and

indicated the positive effects learning strategies. Likewise, [19] indicated that increased reliance on several learning strategies enhances students' performance. However, the finding by [20] revealed that students who used problem-solving approach as well as those who immediately used & combined multiple procedurals were more creative problem solvers than others. Yet, [21] emphasized that organizing strategies, such as selection of information and construction of relations among ideas as strategies of learning Mathematics, among others, have helped students achieve their goals.

Studies on the effect of perceived task difficulty on Math achievement were also revealed differing results. Negative significant effect was evident in the studies by [22], and positive effects of students' perceived task difficulty on their mathematics achievement were also revealed in study by [23].

The researchers have specifically inclined towards investigating the issue related to Mathematics achievement because the researchers have been pressed by unimproved achievement in Mathematics despite the policy reforms undertaken by ministry of education of Ethiopia [16], and due to little emphasis given by researchers to student-related factors rather than instruction-related factors [24]. Hence, the current study aimed to find out effects of students' Mathematics learning strategy and perceived task difficulty on their achievement of Mathematics.

This study uniquely contributes to the field of education and psychology in many ways. The eight dimensions created in Mathematics learning strategy as well as the two dimensions created in perceived Math task difficulty helps to expand our insight of these two constructs. Moreover, there are *theoretical implications* discussed in the later sections in this research report which expands our understanding about environmental factors in the triadic model.

1.2 Objectives of the study

Generally, this study was aimed at investigating effects of students' mathematics learning strategies and perceived task difficulty on their achievement.

Specifically, this study was indebted:

- To find out whether students' Mathematics learning strategy significantly predict their achievement of Mathematics.
- To examine whether students' perceived task difficulty significantly predict their achievement of Mathematics

1.3 Theoretical root

1.3.1 Social cognitive theory. From the perspectives of social cognitive theory of [25] the leaners' behavior is a result of the personal, the environmental and the behavioral determinants, described as triadic reciprocality. The personal factors include students' knowledge and skill, self-efficacy, perception of goals and outcomes. Behavioral factors can include self-observation, learning strategies, self-judgment and self-reaction, whereas, environmental factors involve all the physical and social contexts of the school and classroom. From the perspectives of social cognitive theory, the personal, behavioral, and environmental influence are assumed to be interdependent.

Some proponents like [26] argue that though the three components are integral components of the triadic model, they (personal, behavioral and environmental factors) have not equal weight all the time. In current study, students' knowledge and perception about the Math task difficulty (personal) interacts with his/her selection of Math learning strategies (behavioral), which also determined partly by his/her extent of interaction with students (environmental).

In the current study, students' achievement is the outcome of interplay between employing of learning strategy and his/her perception of task difficulty. Empirically, students' achievement in mathematics is highly a function of strategies employed in dealing with the content [27,28]. The selection and employing of learning strategy is a behavioral component, however, it does not necessarily mean that students rely solely on a specific strategy because it is possible to use more than one learning strategy depending on the context and the content [29]. Hence, it is less arguable that the type of learning strategy the students use and the extent to which they employ it in dealing with the content greatly determine their achievement.

The personal factor, that is, perception of task difficulty in the current case, also expected to affect students' dealing with the content and the resultant achievement. By many students, Mathematics is perceived as a difficult subject [30,31], and this hinders their task engagement in the classroom routines [32] as well as their achievement [12]. Students' Mathematics learning strategy and perceived task difficulty interplay to affect their achievement and achievement related behaviors. It is indicated that students' perception of task difficulty affects their achievement related behaviors [33], and dealing with the task [34], one of which is learning strategy. In the other angle, it is stated by [35] that in employing particular learning strategy effectively, students tend to increase the likelihood of recalling, which by itself, helps students perceive the task as less difficult.

Hence, in the current study, the social cognitive theory has set a theoretical foundation of the overall approach to the problems under consideration.

1.3.2 The value-expectancy theory. The value-expectancy theory of motivation is also another theoretical underpinning of this study. An important point in expectancy value theory is that a student behavior, including proper implementation of learning strategy and having appropriate level of perceived task difficulty, depends on his/her expectancy of attaining an outcome as well as how much he/she values that outcome. Outcome expectation is highly associated with both engagement and achievement [36]. Most often, as a student perceives that the likelihood of success is high, then he/she is likely to engage in classroom routines and the reverse is true if he/she perceives that likelihood of success is low or minimum. This is because individuals' beliefs, values and goals are key reasons of motivation to engage in tasks [37]. Hence, a student is less likely to engage in classroom tasks if he/she fails to find the worthwhileness of the goal, and engagement is an important predictor of achievement [38]. Moreover, a student who perceives that likelihood of success is high is more self-efficacious [25], and he/she less likely perceives the task as difficult task.

Some authors also stressed that even positive outcome, for instance, in the case of current study, to pass the exam, cannot be a reason for a student to engage in task if the outcome is not valued by the student. As stated by [12], a student doesn't merely consider the immediate result, but also tends compare the ultimate goal to be achieved with the cost he/she is going to incur while striving towards the goal. This is because it clear that engagement, among other things, is highly affected by motivation to undertake tasks [39], and, individuals' beliefs, values and goals are key reasons of motivation [40]. Due to these patterns of relationships, in the current study, the value-expectancy theory of motivation is another theoretical foundation for approaches.

1.4 Conceptual frameworks

Based on the above theoretical bases, the researchers have hypothesized that students' perceived Math task difficulty and Math learning strategy influence their achievement. The hypothesized relationships among the three variables treated in this study are stated in the following conceptual framework as indicated in Fig 1 below.

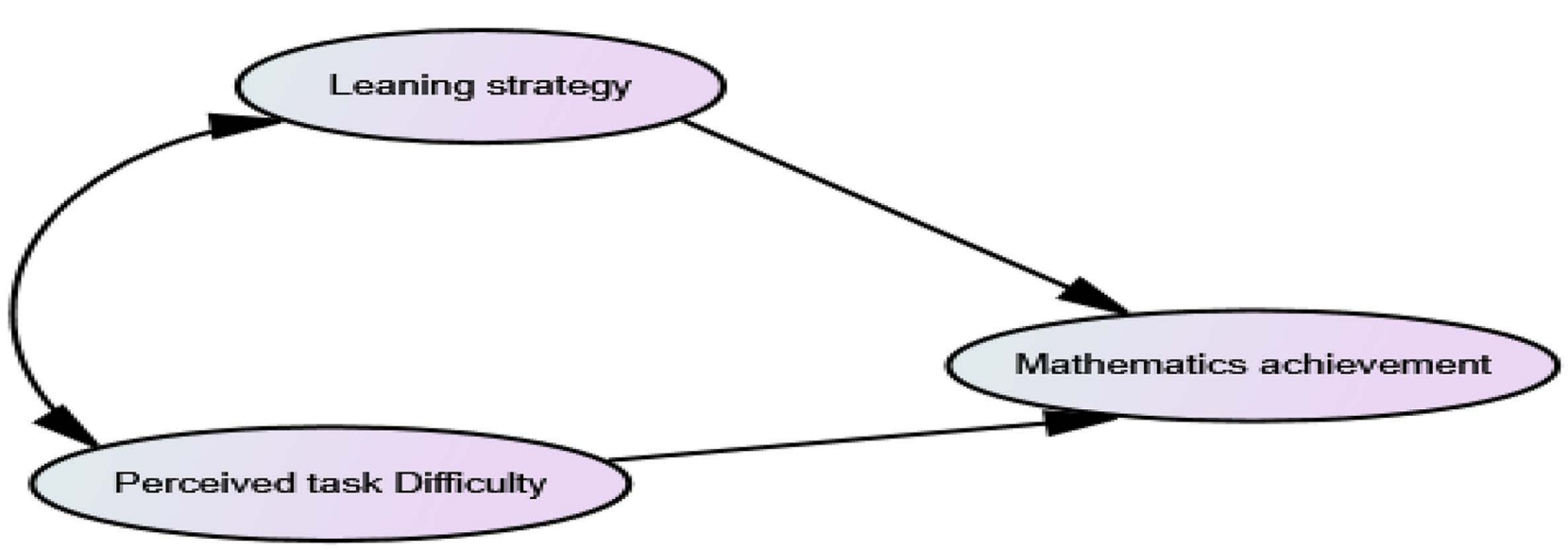

Fig 1. The conceptual frameworks as conceived by the researchers.

https://doi.org/10.1371/journal.pone.0337115.g001

2 Methods

2.1 Research paradigm, approach and design

This study relied on post-positivism paradigm or view of the world. It further employed a quantitative approach, and a correlational design. As stated by [41] correlational studies can be either prediction studies where the researcher is interested in using one or more predictor variables to project performance on one or more criterion variables or relationship studies, where the researchers undertake study to explore the relationships between measures of different variables to gain a better understanding of factors that contribute to more complex characteristics. Since the current study involves prediction on the outcome variables, the correlational design better suits to the current scenario.

2.2 Population, sample and sampling techniques

The population of this study was students of 7 public secondary schools in the town administration, all enrolling students from grades 9–12. Among these, one school was selected through simple random sampling. Then, two grade levels, namely, 10th and 11th grades were selected purposively. The rationale behind was students' prepare themselves for national examination in grade 12 which might have changed their engagement and in grade 9, they were promoted from grade 8 and might have been struggling with new setting, where in both cases students are less likely to exhibit usual achievement related behaviors. In these selected grade levels in the school, there were a total of 2893 (two thousand eight hundred ninety three) 10th and 11th grade students enrolled in the academic year 2024/25. To determine the sample size, the researchers relied on the formula developed by [42] as:

$$n = \frac{N}{1 + N(e)^2}$$

Where 'n' is the sample size, 'N' is the population size, and 'e' is the level of precision. The researchers assumed 95% confidence level and 0.05 level of error.

$$n = \frac{2893}{1 + 2893(0.05)^2} \quad \text{Nearly, } 351$$

Hence, 351 sampled students and additional 10% of reserve respondents were made to fill the questionnaire. Accordingly, the data was collected from 386 respondents using systematic random sampling. Among these 375 (97.1%), have returned the paper back. In the first round screening, 12 papers were identified as incomplete and 8 papers were rejected because they were detected as univariate and multivariate outliers. As 355 cases passed the cleaning process, four cases were rejected through random sampling and the 351 cases were finalized for main analysis.

2.3 Instrument development process

Two variables, namely, Mathematics learning strategy and perceived task difficulty were measured using questionnaire, whereas, students' achievement was measured by the tests that were prepared after due discussion and consensus with Math teachers in selected grade levels about relying on table of specification to develop items. In order to maintain uniformity of tests, after due discussion of researchers with the subject teachers, equal number items, equal forms of items with regard to objective-subjective dichotomy, with equal number of chapters covered in the test, were prepared and administered. However, the tests were prepared, administered and scored in the manner that doesn't jeopardize the regular instructional process. Due to this, it took the teachers about two weeks to complete scoring all essay and objective item types in the test. Then the teachers reported it to record office, and researchers have recollected it.

The variable Math learning strategy was measured adapting the tool used by [43]. The variable perceived task difficulty was measured by adapting the tool developed and used by [44].

2.4 Reliability and Validity Tests

The content validity index has been conducted by researchers, where items have been rated for 'clarity' and 'relevance' by 6 psychology experts ranging from the level of lecturer to associate professor. Accordingly, for both clarity and relevance, the scale content validity index (scale-CVI) appeared above 0.9 for both scales of learning strategy and perceived task difficulty. However, amendments have been undertaken for 7 items in learning strategy scale and 2 items in perceived task difficulty after the suggestion by the experts.

With pilot data, convergent validity has also been carried out. To do so, the average variance extracted (AVE) was computed by summing up squared correlations (R^2) of items in each dimension and dividing them by number of items as suggested by [45]. As to Fornell and Larcker, cited in [45], average variance extracted (AVE) above 0.5 is a threshold to denote that there is convergent validity among dimensions which has been met in the pilot data of the current study for dimensions in both scales.

Cronbach alpha was applied on the pilot study to assure reliability. In pilot study, which was conducted on 140 students in secondary schools out of the main study area, 46 out of 55 items for the scale of Math learning strategy as well as 9 items for perceived task difficulty, were proved to have item-total correlation above 0.3, which was a cut point used to reject items as suggested by [46]. The total Cronbach alpha appeared 0.897 for learning strategy and 0.707 for perceived task difficulty.

With regard to discriminant validity, it is stated by [45] that the shared variance between two dimensions must be lower than AVE of either of the dimensions. In the current study, AVE result appeared 0.53 for *organization*, 0.54 for *elaboration*, 0.55 for *time management related effort*, 0.51 for *repeating*, 0.57 for *managing learning environment*, 0.69 for *peer learning*, 0.51 for *repeating*, and 0.501 for *exercise and effort*. The computed results of shared variance ranged from 0.09 of between *organization* and *managing learning environment* to that of 0.28 between *time management related effort* and *exercise and effort*. Hence, lack of shared variance result between any two dimensions that exceed their respective AVE denotes that the discriminant validity among dimensions of the construct *learning strategy* is achieved.

Likewise AVE appeared 0.59 for *pre-requisite related difficulty* and 0.501 for *task related difficultly.* However, there shared variance between the two dimensions appeared 0.175 which denoted that there is discriminant validity between the dimensions of perceived task difficulty.

2.5 Factor analysis

2.5.1 Exploratory factor analysis. In the main data, prior to EFA, the assumption related to sample size has been tested and proved in addition to item-total correlations, which was already maintained in pilot study. It is suggested by [47] that a minimum sample size for exploratory factor analysis is 150+which was met in the current sample. Consequently, 46 items for learning strategy and 9 items for perceived task difficulty have been finalized for EFA.

Using AMOS with in SPSS version 25, the principal component analysis (PCA) with varimax rotation was run. The researchers have employed the varimax rotation because as to [48], it uses a mathematical algorithm that maximizes high and low value factor loadings and minimizes mid-value loadings, which was needed in the current study to reduce dimensions. Moreover, the researchers have suppressed loadings less than |.40| because [49] have pinpointed that loadings of |.40| or greater are taken as high.

For the scale of Math learning strategy, it has been revealed that the KMO of 0.924 and Bartlett's test of Sphericity (Chi-square = 12733.663, df = 1035; $p < 0.01$) indicating that further analysis was suitable which was done as follows.

As indicated in Table 1, the result of PCA with iterated Varimax rotation run for Math learning strategy, has pinpointed the presence of 8 components with Eigen value above 1 by explaining 71.7% of total variance accounted for by the

Table 1. Rotated component matrix for math learning strategy.

	Component							
	1	2	3	4	5	6	7	8
2 I compile short summaries of the most important contents as a mnemonic aid.			.651					
3 I go over my notes and structure the most important points.			.796					
4 I try to order the subject matter in a way that makes it easy for me to remember.			.726					
5 I compile a summary of the main ideas out of my notes, the script or other sources			.829					
6 I underline the most important parts in my notes or in the text			.676					
7 For bigger amounts of subject matter, I find an arrangement that mirrors the structure best			.777					
8 I assemble important terms and definitions in my own lists			.740					
9 I try to find connections to other subjects or courses.		.824						
10 I think of practical applications of new concepts.		.805						
11 I try to relate new terms or theories to terms or theories I already know.		.634						
12 I visualize new issues.		.710						
13 In my mind I try to connect newly learnt facts to what I already know.		.794						
14 I think of practical examples for certain curricular facts.		.736						
15 I relate what I am learning to my own experiences.		.706						
16 I wonder if the subject matter is relevant to my everyday life		.593						
17 I imprint the subject matter from the lecture on my memory by repeating it.				.806				
18 I read my notes several times in a row.				.668				
19 I learn key terms by heart in order to remember important facts better in the exam.				.763				
20 I commit a self-compiled compendium to memory.				.815				
21 I read the text and try to recite it at the end of each paragraph.				.654				
22 I commit rules, technical terms, or formulas to memory.				.638				
23 I learn the subject matter by heart using scripts or other notes.							.652	
24 Whenever I have planned a certain workload, I make an effort to master it.							.791	
25 I make an effort even though the subject matter may not suit me well.							.769	
26 I do not give up even though the subject matter is very difficult and complex.							.716	
27 I work late at night or at the weekends if necessary								.585
29 Before exams I take the time to go over all the subject matter again.								.788
30 I take more time for learning than most of my fellow students.								.742
31 I work until I am sure to pass the exam well.								.674
38 I fix the hours I spend daily on learning in a schedule.	.837							
39 Before each study period I appoint the duration of my work.	.843							
40 I work according to a schedule.	.837							
41 I work in a place that makes it easy to concentrate.						.619		
42 I design my work environment in a way that I am distracted as little as possible.						.652		
43 When studying I make sure that I can work uninterrupted						.822		
44 My workplace is designed in a way that makes it easy to find everything.						.851		
45 At my desk I have the most important papers within reach.						.810		
46 I work on tasks together with my peer students.					.834			
47 I take my time to discuss the subject matter with other students.					.760			
49 I make other students ask me questions on the subject matter and ask them questions too.					.801			
50 I turn to help from others when I have serious problems in understanding something.					.860			
51 When I am not sure about something, I ask a fellow student for advice.					.802			
52 If I find considerable gaps in my notes, I turn to fellow students.	.831							
53 I search for explanatory material if certain facts are not completely clear.	.825							
54 I look for missing information in different sources, e.g., the Internet, textbooks, or journals.	.650							

(Continued)

Table 1. (Continued)

	Component							
	1	2	3	4	5	6	7	8
55 When my notes are incomplete, I use additional sources.	.792							
Number of items	7	8	7	6	5	5	4	4
Eigen values	16.04	3.28	2.75	2.55	2.44	1.93	1.60	1.25
Cronbach alpha	.818	.824	.706	.607	.836	.664	.640	.690
% of variance explained	35.54	8.15	6.76	5.58	5.42	4.48	3.23	2.52

https://doi.org/10.1371/journal.pone.0337115.t001

components. The components from 1–8 were named as *referencing or reference use*, *elaboration, organization, repeating, peer learning, managing learning environment, exercise and effort,* and *time management with effort*.

The naming of the dimensions was rooted partly on the naming used in previous study by [43]. It also partly based on suggestions by [49], where it is indicated that naming of the dimensions need to be based on the nature of items and selecting the name in to which the loaded items conceptually fit together.

The communality of variables range from 0.33 to 0.901 for items 54 and 50 (both are in Table 1) respectively, which is good in that as to [50], the communlaity values exceeding 1 can casue convergence problems.

For the scale of perceived task difficulty, in the first round, there indicated that the KMO of 0.826 and Bartlett's test of Sphericity appeared significant (Chi-sqaure = 659.891, df = 36; $p < 0.01$). However, item 4 ('There remains an insecurity about the solution to Math task.) was not loaded to any factor with suppressing loadings below 0.40. Due to this, the researchers have run the PCA again by excluding item number 4. In this round, KMO of 0.791 and Bartlett's test of Sphericity (Chi-sqaure = 1194.199, df = 28; $p < 0.01$) indicated that further analysis was permitted.

In Table 2, the result of PCA with iterated Varimax rotation run for perceived task difficulty has revealed the presence of 2 components with Eigen value above 1 by explaining a total of 64.39% of total variance. The first dimension was named as 'Prerequisite related difficulty', whereas, the second dimension was named as 'Task related difficultly'. The communality of variables range from 0.301 to 0.814 for items 7 and 9 (both are in Table 2) respectively, which was proved good.

Table 2. Rotated factor matrix for perceived task difficulty.

Items	Components	
	1	2
I have undertaken similar tasks in the past, and hence, I am familiar with the design of Math task.	.667	
I had too little information, while completing Math task.		.780
There were multiple possible ways to come to a solution for Math task.	.834	
I found Math task a difficult one.	.753	
The Math task is not completely unknown to me because I have undertaken similar tasks in the past.	.825	
I could have solved the Math task in a different way than the way I have used.		.539
I found Math task a complicated one.		.530
In doing Math tasks, information resources were difficult to access		.914
Number of items	4	4
Eigen values	3.64	1.50
Cronbach alpha	.840	.744
% of variance explained	45.53	18.86

https://doi.org/10.1371/journal.pone.0337115.t002

Like that of the construct learning strategy, the naming of the dimensions in PTD was rooted on suggestions by [49] by properly identifying the phrase in to which the loaded items conceptually fit together.

2.6 Confirmatory factor analysis (CFA)

2.6.1 The initial measurement model. The confirmatory factor analysis was computed by starting with measurement model using AMOS version 25. The result of fit indices in initial measurement model indicated that the model is not a good model and doesn't fit to the data. It is suggested by [45] and [46] that the results of six of these indices, that is, GFI, AGFI, IFI, CFI, NFI, RFI and TLI need to be above 0.9, whilst, that of RMSEA expected to be below 0.05. Whilst only four indices, that is, GFI, CFI, IFI and CMIN/DF tended to meet the threshold level, the rest 4 indices were failed to meet the required threshold level, which implies poor model fit to the data in this phase.

Despite the presence of some indices indicating the model unfit, same result indicated that all paths in the measurement model were found to be statistically significant with the critical ratio test greater than ± 1.96 at $p<0.05$. Due to this the researchers have decided to take modification actions by correlating the 2 error terms of observed variables to improve the measurement and the indices in resultant new model was presented in Table 3 as follows.

The result of modified measurement model in Table 3 has clearly indicated that the model fit to the data across all the fit indices χ^2 (N = 351, df = 40) = 118.939, $p<.05$). As indicated in Table 3 above, the result indicated that all the model fit indices were found to fit their respective threshold level with $p<0.05$ following the improvement through correlating error terms. The maximum likelihood estimates of the unstandardized and standardized regression coefficients have pointed out that all the path coefficients in the modified measurement model were found significant at $p<0.05$.
Finally, the model has confirmed that the unstandardized and regression weights of 2 dimensions of perceived task difficulty and 8 mathematics learning strategies were significant with ±1.96 at $p<.05$. It also revealed that standardized regression weights of all 10 dimensions in the model significantly represented by their respective latent variables of perceived task difficulty and Mathematics learning strategies to predict the dependent variable at $p<0.05$. The variances of dimensions explained by their respective latent variables in Math learning strategy range from 29.9% for *managing learning environment* to 56.5% for *exercise and effort* at $p<0.05$. With regard to perceived task difficulty, it ranged from 60.3% for *pre requite-related perceived task difficulty* to 71.7% for *task related perceived task difficulty*.

2.7 Data collection process

The main data was collected within two weeks from mid to late October, 2024 (14th October to 25th October, 2024) in the selected school. The questionnaire was administered in classroom after clarification on overall feature and description on anonymity with the help of data collectors and colleagues and with the coordination of one representative among researchers.

Table 3. Fitness indices of the modified measurement model.

Criteria	Obtained values	Needed threshold	Threshold meet?
Relative chi-square (CMIN/DF)	2.973	< 5	Yes
Root mean square error of approximation (RMSEA)	.048	< 0.5	Yes
Comparative fit index (CFI)	.939	>0.9	Yes
Adjusted goodness of fit index (AGFI)	.909	>0.9	Yes
Normed fit index (NFI)	.912	>0.9	Yes
Goodness of fit index (GFI)	.945	>0.9	Yes
Tucker-Lewis index (TLI)	.917	>0.9	Yes
Incremental fit index (IFI)	.940	>0.9	Yes

https://doi.org/10.1371/journal.pone.0337115.t003

2.8 Ethics approval

Data gathering instruments and data collection process undergone through ethical approval by host institution's ethical clearance office with the project code 009 and the protocol code 001952 on 23rd July, 2024. Further, during data collection process, the respondents were provided with the written consent and only those for whom willingness has been secured were included in the study.

2.9 Methods of data analysis

This study has employed both descriptive and inferential statistics. SPSS version 25 and AMOS version 25 were software types used for data analysis. To present the status of students' mathematics achievement mean, maximum and minimum score were involved. To find out effect of perceived task difficulty on achievement in Mathematics, and to find out effect of learning strategy on achievement in Mathematics, structural equation modeling was employed.

3 Results and interpretations

The result is presented in different sections as demographic characteristics of participants, descriptive statistics on two independent variables as well as results addressing two specific objectives.

3.1 Demographic characteristics of participants

The demographic characteristics of participants is presented in Table 2 follows

As indicated in Table 4 above, 51.9% of participants were males, whereas, 48.1% of them were females. Among the participant students, 47.6% were 16 and 17 years old, 42.2% were aged from 18 to 19 years and the rest 10.3% were aged above 19 years. Moreover, 60.7% of participants were grade 10 students and 39.3% of them were grade 11 students.

3.2 Descriptive statistics

3.2.1 Descriptive statistics on students' Mathematics achievement across grades. As indicated in Fig 2, for grade 10, the mean, minimum and maximum scores were 59.73, 27 and 82 respectively for males, whereas, 57.89, 38 and 81 respectively for females, which indicates that males surpassed females in maximum and mean scores. The result also indicated that for grade 11, the mean, minimum and maximum scores were 58.01, 27 and 80 respectively for males, and 56.65, 35.5 and 82 respectively, for females, which indicate that females surpassed males in maximum and minimum scores whereas, males scored better than females in average.

From the result in Fig 2, it can also be inferred that males achieve better than females in average in both classes. This may be possibly because of the sociocultural barriers where females at secondary school level in the study area do highly share home duties with their parents than males.

Table 4. Demographic characteristics of participants.

Variables		Frequency	Percent
**Sex**	Male	182	51.9
	Female	169	48.1
Age	16- 17 years	167	47.6
	18-19 years	148	42.2
	> 19 years	36	10.3
Grade	Grade 10	213	60.7
	Grade 11	138	39.3

https://doi.org/10.1371/journal.pone.0337115.t004

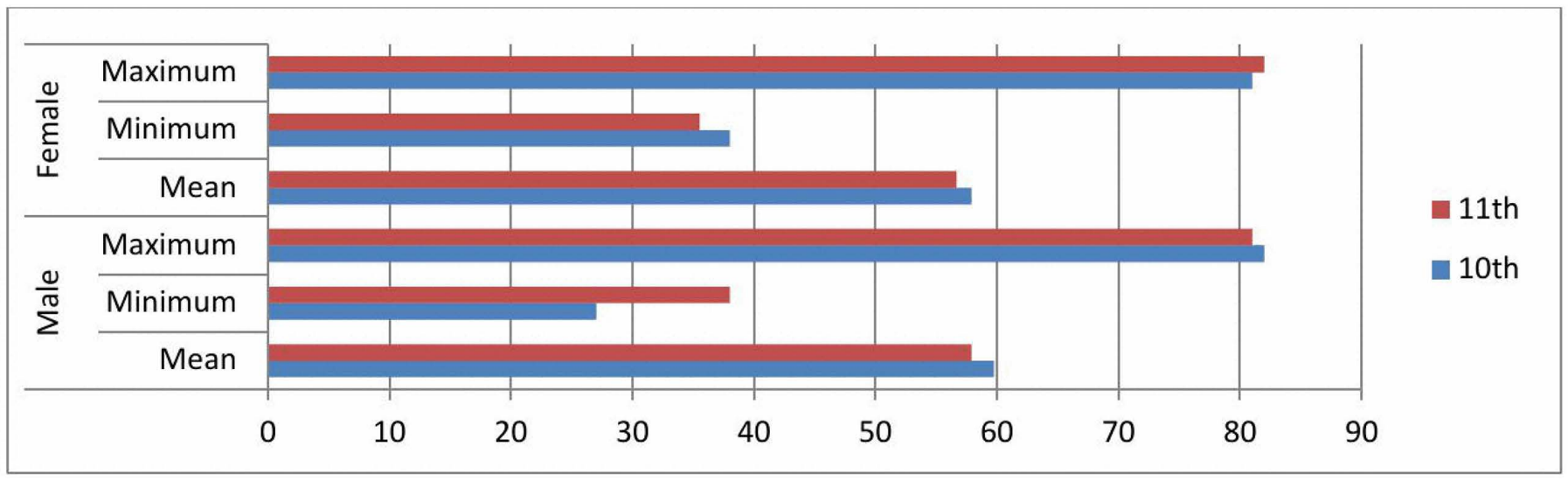

Fig 2. Descriptive statistics on students' mathematics achievement teacher-made tests. Source: The main research data 2024, N = 351.

https://doi.org/10.1371/journal.pone.0337115.g002

3.2.2 Descriptive results on dimensions of learning strategy. As indicated in Table 5, peer learning has highest mean score (4.01) followed by time management related effort (3.4), whereas, repeating has the lowest mean score (2.42), followed by managing learning environment (2.48).

In the current education system of Ethiopia, at secondary school level, peer learning is highly advocated and practiced using mixed grouping where five students are assigned to one group with the hope that it can bring changes on students' performance [51], and its theoretical root is the social constructivist theory of Vygotsky [52]. However, the relative low scores in managing learning environment and reference use is annoying as learning environment need to be properly managed as suggested by [53]. Likewise, reference use is also a critical concern in that, as stated by [54], a teacher needed to help students unfold their potentials by leading them to dig out knowledge and facts from books and reference materials, which is of low practice in the current study.

3.2.3 Descriptive results on dimensions of learning strategy. As indicated in Table 6, the dimension *task related difficulty* has more or less similar mean score (3.43) that of *prerequisite-related task difficulty* (3.45). This indicates that the students generally perceive mathematics tasks as difficult either by thinking that they lack pre-requisite skill or perceiving that the task itself is difficult to try and engage in.

3.3 The effect of students' perceived task difficulty and Mathematics learning strategies on their mathematics achievement

3.3.1 Structural model. Using the issues resolved in the modified measurement model as bases, the researchers have developed structural model for the study using dimensions confirmed by the measurement model.

Table 5. Descriptive results on dimensions of learning strategy.

Descriptive statistics	Organization	Elaboration	Repeating	Exercise and effort	Time management related effort	Reference use	Managing learning environment	Peer learning
Mean	3.27	3.25	2.42	3.32	3.40	2.81	2.48	4.01
Median	3.14	3.12	2.0	3.5	3.5	2.83	2.4	4.0
Standard Deviation	0.81	0.88	1.11	0.93	0.94	1.38	1.11	1.07

https://doi.org/10.1371/journal.pone.0337115.t005

Table 6. Descriptive statistics on dimensions of perceived task difficulty.

Descriptive statistics	Task related difficulty	Prerequisite related difficulty
Mean	3.43	3.45
Median	3.75	3.5
Standard Deviation	0.85	0.92

https://doi.org/10.1371/journal.pone.0337115.t006

As indicated in Fig 3, two latent variables, that were, perceived task difficulty, Mathematics learning strategy; the corrected error terms and one observed variable, that was, Mathematics achievement were tested for their relationships in the structure of the path model to check how much of the dependent variable is predicted by the independent variables.

The SEM analysis made using AMOS 25 to test the entire fit of the model using the eight indices, like that of modified measurement model, revealed that the model set to fit to the data across all the fit indices χ^2 (N = 351, df = 40)= 118.939, $p < .05$. In the model, the fit indices of data, revealed that GFI was.945, the AGFI was.909, the IFI was.940, the CFI was.939, the NFI was.912, the TLI was.917, the RMSEA was 0.048, the CMIN/DF was 2.973, all indicated that the hypothesized structural model fits to the data, and confirming that the structural model is valid enough to make causal relationships among variables.

The result of the measurement model in Table 7 above indicated that in the model, total of 10 dimensions were evident, and were significantly represented by their respective latent variables. The result ranges from.106 for difficulty related

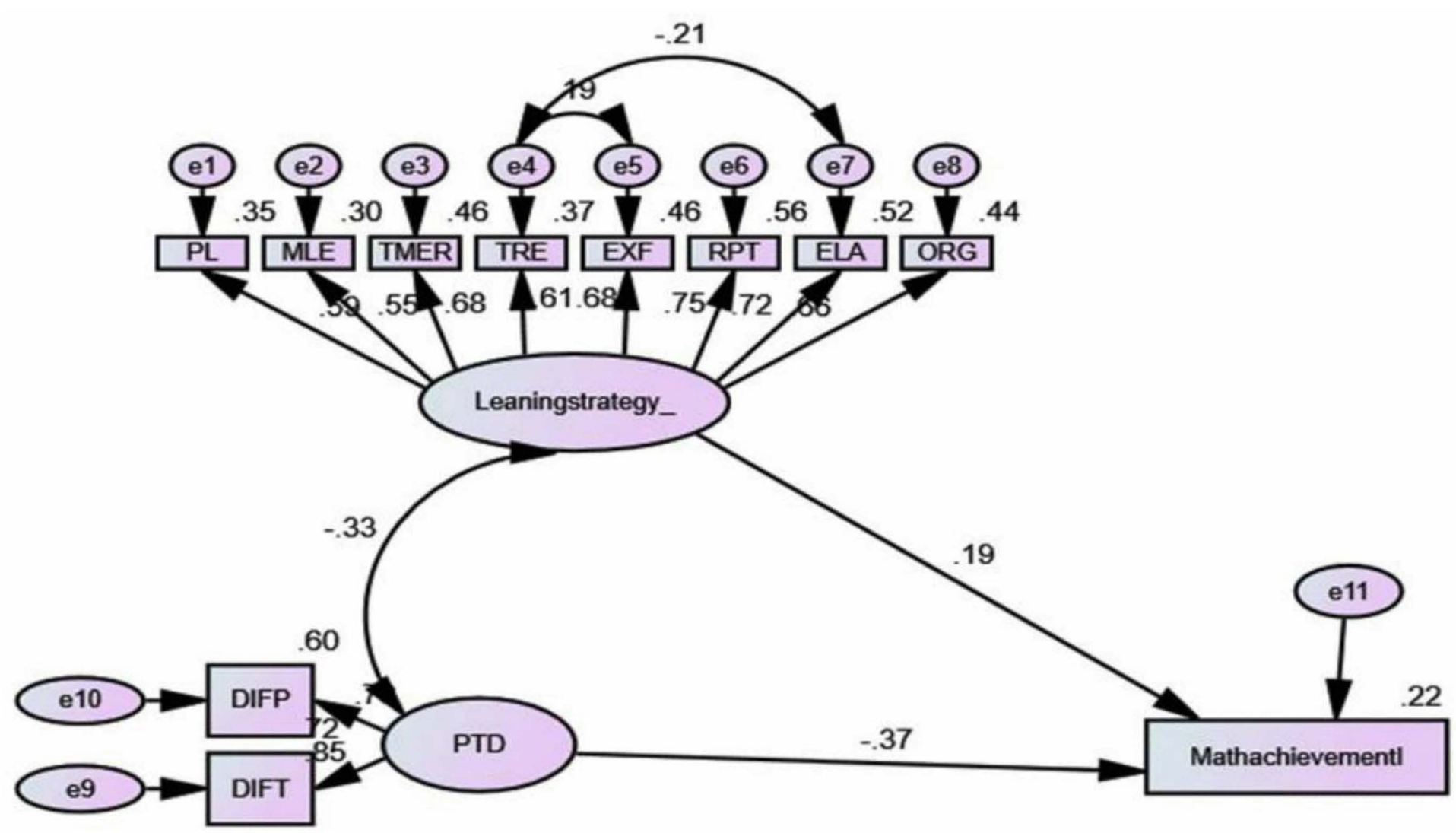

Fig 3. The structural model of the study. Source: The main research data 2024, N = 351. RPT = *referencing or reference use*, ELA = *elaboration, ORG = organization, RPT = repeating, PL = peer learning MLE = managing learning environment, TMER = time management with effort*, EXE = *exercise related effort, PTD = perceived task difficulty, learningstrategy_ = Learning strategy, MathachievementI = Achievement in Mathematics.*

https://doi.org/10.1371/journal.pone.0337115.g003

Table 7. Unstandardized and standardized regression weights of dimensions in the measurement model.

Variables			Estimate	S.E.	C.R.	P	Label
Organization	←	Learning Strategy (LS)	1.000				.688
Elaboration	←	LS	1.237	.106	11.686	***	.698
Repeating	←	LS	1.025	.081	12.602	***	.760
Exercise and effort	←	LS	.684	.057	11.956	***	.716
Time related effort	←	LS	.584	.055	10.567	***	.625
Time management and references	←	LS	1.119	.097	11.566	***	.690
Managing learning environment	←	LS	.587	.061	9.600	***	.564
Peer learning	←	LS	.745	.072	10.325	***	.610
Task related perceived Difficulty	←	Perceived task difficulty (PTD)	1.000				.574
Prerequisite related perceived Difficulty	←	PTD	1.961	.506	3.879	***	.106

https://doi.org/10.1371/journal.pone.0337115.t007

Math task difficulty to.760 for *repeating* all found to be significant, indicating that the observed variables have significantly explained their respective latent constructs.

3.3.2 Regression Coefficients on the effect of students' Math learning strategy and perceived task difficulty on their Math achievement. The following table presents the regression weights on the effect of perceived task difficulty and Mathematics learning strategies on their Mathematics achievement.

The result in Table 8 indicates that unstandardized regression weights of perceived task difficulty and mathematics learning strategies were found to be significant with critical ratio tests greater than ±1.96 at $p < .05$. The standard regression weights coefficients of perceived task difficulty and Math learning strategy, that is, −.374 and.186 respectively indicated that the dependent variable was significantly predicted by the independent variable with $p = 0.00$. The maximum likelihood estimates of the standardized and unstandardized regression coefficients also confirmed that all path coefficients in the structural model were found significant at $p < 0.05$.

As the result in Table 8 and in the path model in Fig 3 indicated, that perceived task difficulty has significant effect on students' achievement in Mathematics. The result in Table 8 indicates that the standardized regression weights of perceived task difficulty on students' mathematics achievement was statistically significant ($\beta = -.374$, $p < .05$).

As the result in Table 8 and the path model in Fig 3 indicated, students' mathematics learning strategy has significant effect on their Mathematics achievement. The result in Table 3 indicates that the standardized regression weights of students' mathematics learning strategy on their mathematics achievement was statistically significant ($\beta = .186$, $p < .05$).

The value of R^2 revealed that 22% of variance in students' Mathematics achievement was predicted by both perceived task difficulty and Mathematics learning strategy, whereas, 78% variance in mathematics achievement is not predicted by the independent variables. The amount of variance not explained by the combined independent variables is high. However, since the study treated only two independent variables, the explained amount of variance in the dependent variable explained can be taken as high. Hence, it indicates that the model is good model and the independent variables in the model have well explained the dependent variable compared to their number.

3.4 Discussions

In the current study, the result revealed that Math learning strategy of students have significant effect on their achievement. Across the skimming of previous studies on the effect of Math learning strategy of students on their achievement, results pinpointed the two variables were conceptually and causally related in various ways. [55] stated that as one of learning strategy, collaborative learning strategy supports students' specific content based knowledge and enhances their problem solving competencies. This is likely due to the fact that collaborative learning can enhance social acceptance of individuals, as it strengthens group cohesion through which they learn from each other and improve their achievement [56]. Time management related effort is designated as learning strategy and it is stated that as a student devote more time and exert more effort, he/she is likely to enjoy with academic success [57].

Empirically, like the current finding, the study by [58] revealed that learning strategies have significantly predicted students' academic achievement. However, in several other studies, there found to have variations in the extent of effects of students' learning strategies on their achievement. With regard to this, the study by [59] indicated that those students who use memorization strategies have achieved lower than those who used other strategies. In the study by [18], the control

Table 8. Standardized and unstandardized coefficients of perceived task difficulty and Mathematics learning strategies on mathematics achievement.

Variables			Unstandardized estimates				Standardized
			Estimate	S.E.	C.R.	P	
Mathematics achievement	←	Leaning strategy	.645	.202	3.195	.001	.186
Mathematics achievement	←	Perceived task difficulty	−1.059	.183	−5.781	***	−.374

https://doi.org/10.1371/journal.pone.0337115.t008

strategies contributed to higher achievements in Mathematics, whereas, the study by [60] pointed that employing computer and interactive learning strategies lead to better achievement. Still, the result of study by [61] has shown that making the study place quite and convenient positively affects students' achievement. As managing learning environment was treated as one of mathematics learning strategies, the effect of change in learning environment contributes to achievement, the finding directly supports the stand that learning strategies have positive effect on students' achievement.

The study on the effect of learning strategies on students' Mathematics achievement also have dealt about the role of number of strategies used as well as vice versa causation between the two variables. Evidences pinpointed that using as many strategies as possible to internalize the content someone dealing with, has significant positive effect on students' achievement. It has been indicated by [62] that low achievers do not use many strategies. Another pattern of relationship is that achievement behavior of learners tended to deter students' selection of learning strategies. The study by [63] indicated that students who achieve high relied on deep learning strategies or tended to eclectically use several strategies.

Generally, several studies on the effect of students' Mathematics learning strategies on their achievement were conducted but raised differing effects than the current finding, where those few which replicated the current finding have also spatial and methodological variation. Hence, the current finding is valuable enough to contribute to the body of knowledge in the area.

Unprecedented number of scholars argues that perceiving the task as difficult negatively contributes to academic performance. A student who perceives the task as difficult is likely to receive less reinforcement as he/she refrain himself/herself from on-task behaviors [64]. This may be because as the child thinks the task is difficult, he/she has lower levels of interest ([65], lower levels of affect [66], lower expectancy of success and consequent lower cognitive engagement [67], all of which are key factors in achievement.

In contrary, others argue that increasing task difficulty is a key factor in enhancing performance. [68] have a stand that effort and on task behavior are likely to increase as perceived task difficulty increases. Still, [69] has a stand that only moderate tasks have relatively better positive effect on students' performance because too easy and too difficult tasks are likely to decrease effort and motivation.

In the current study, it is also revealed that perceived task difficulty had significant negative effect on their mathematics achievement. Previous studies on the cause-effect relationship between the two have revealed differing results. The study by [70] indicated that task difficulty has negative effect on students' performance in Mathematics, which partly replicated the current finding but with differing tools. Still, considerable number of studies affirm that the negative effect of perceived task difficulty on students' achievement was evident [71]. In line with this idea, the role of myths which emanate from the perception of math is difficult has also been negative effect on many students' achievement. The study by [72] indicated that low achievement was evident by those who thought themselves as slow-thinkers and consequently, students who thought that achieving well in Mathematics was for fast-thinkers have achieved lower in tests.

However, other studies pinpointed that this causal relationship between the variables is not always comes similar. The stand that increasing the task difficulty might enhance students' Math achievement has also been supported by the finding by [11,73]. Stressing this, [74,75] stated that the moderate levels of perceived difficulty may boost performance while high levels of difficulty are likely to lower it.

To sum up, though the current finding on the effect of perceived task difficulty on students' mathematics achievement was related with some previous findings, still several studies has gave differing result that inspires the essentiality of medium level of difficulty than mere negative effect of perceived task difficulty on students' Mathematics achievement.

4 Conclusion, implications and recommendation

4.1 Conclusion

The latent variables treated in this study, that is, students' mathematics learning strategy, and perceived task difficulty have significantly predicted their Mathematics achievement. This points that helping students perceive task

as easy, added to proper application of organization, elaboration, repeating, exercise and effort, time management related effort, managing learning environment, peer learning, and referencing and time management' boost learners' achievement.

4.2 Practical implications

The result indicated that learning strategy was found to be significant positive predictor of students' achievement. This is to mean that through employing organization, elaboration, repeating, exercise and effort, time management related effort, managing learning environment, peer learning, and referencing', learners' achievement of mathematics will be boosted. Due to this, it is recommendable that Mathematics teachers should help students to rely on one and/or another type of learning strategies while dealing with contents.

Peer learning is an important area of emphasis in the context of Ethiopian secondary schools nowadays. In this study, it has been revealed that peer learning is highly practiced among students (3.98) followed by time management related effort (3.40), which exceeded cognitive and metacognitive strategies such as organization, elaboration and repeating. Moreover, the result also indicates that cognitive strategies are better practiced by students. However, relatively low practice of reference using strategy (2.81) might have contribution for low effect of Mathematics learning strategy on their achievement compared to that of perception of task difficulty. Hence, adequate provision of reference materials and follow up by school administrative bodies and subject teachers whether or not students use them is essential.

It is also vital to enhance students' practice on *managing learning environment* strategy (2.48). Actually, in secondary school level in Ethiopia, the teacher takes lion's share in managing learning environment as school and classroom level. However, in its wider sense, learning environment encompasses any area where a student tends to duly study, like home, library, laboratory and demonstration sites, and some of these, a student himself/herself is responsible for managing. Proper role of student in managing learning environment is not only a key to achieve better, but also is a key to properly implement other strategies such as organization, elaboration, reference use because implementation of them also needs proper managing of learning environment. Hence, students need to be guided by teachers regarding how to properly manage learning environment. Moreover, school administrative bodies need to take initiative in directing laboratory technicians and librarians to avoid factors hindering students' proper management of learning environment like, avoiding noises, arrangement of seats, books and resources.

It is evident that one of the dimensions in perceived task difficulty was prerequisite related task difficulty. It can be inferred from the result that students perceive the task as difficult partly because they thought that they lacked prerequisite skills. Hence, the researchers recommend that curriculum experts need to assure incorporation of prerequisite related contents prior to each of the new content rather than considering that mere vertical integration of contents at textbook level as adequate. It is also recommendable that mathematics teachers emphasize on proper implementation of formative assessment for the very purposes of collecting data on 'where students are?' and 'what prerequisite skills do they lack?' is vital. This is because of the fact that using the result of formative assessment, the teacher can start from where students really are and understands what need to be flashed on students' mind prior to dealing with the lesson's content which can greatly lower the pre-requisite related outcome expectation. Moreover, practically, the perception of task difficulty can be improved through social scaffolding, which as suggested by [76], is a means to help students benefit from the afforded opportunities to engage in and to learn more meaningfully.

In addition, the higher mean score was observed on task-related difficulty than prerequisite related perception of difficulty, which demands much work on Mathematics teachers in helping students perceive Math tasks as easy and attainable. This is possible through proper implementation of inductive-approach while giving exercises where students' better achievement in lower-order tasks can contribute to boost confidence and to increase effort which are key factors in achievement of mathematics.

4.3 Theoretical implications

It seems good that the result in Table 5 indicated that students rely more on relatively higher-order form of cognitive and metacognitive strategies, such as organization and elaboration than *repeating*, which is a rote learning. However, students less tended to rely on managing learning environment. This may be either due to the fact that many of learning environments in instructional process are managed by other bodies than student himself/herself like teacher, lab-technicians and librarians, or due to the fact that students remain with little role to play in managing the outside-school learning environments like the worthwhileness of the 'good result' itself in post COVID-19 Ethiopia, job opportunities after completion of schooling and the life condition incurred by the graduated and employed counterparts, which is not awesome nowadays in Ethiopia, as to [77]. Due to this, in the triadic model, particularly, in the environmental factors' category, it is unequivocally essential to go far beyond immediate classroom and school environments and it is vital to equally emphasize on the outside social conditions.

4.6 Limitations and future directions

Due to homogeneity of the study population, added to budget constraints, this study has been geographically delimited to the selected secondary school, which affects its generalizability to the whole Ethiopian secondary schools. Due to this, interested researchers need to undertake the further study in the same issue by including additional number of schools, by employing mixed design and by including mediating variables.

It is also essential to include other variables, for instance, investigating the mediating roles of self-efficacy and/or academic engagement in the relationship between the independent variables such as learning strategy and perceived task difficulty and the dependent variable mathematics achievement. Moreover, socioeconomic and cultural aspects need to be given due consideration during translation of items and generalization of findings when conducting further studies.

The perceived task difficulty might have role on enhancing or lowering both the self-efficacy and academic engagement of students, which by itself, can affect achievement. Likewise, learning strategy might affect behavioral and cognitive engagement, and change is engagement is likely accompanied by changes in achievement as proved by many studies. Moreover, as a student tend to use more references, use elaboration, better manage environment, increase effort and the like, he/she definitely engaging in academic tasks, which can ultimately enhance achievement. Hence, investigating the mediating role of self-efficacy as well as that of academic engagement can add the stalk of knowledge to the field of educational psychology and also can deepen our understanding of the relationship between dependent and independent variables treated in the current study. With the intention to enhance our understanding of the relationships among the variables in this study, one can also add teacher-related variables such a teaching methodology, teachers' intimacy with students and teacher's subject matter knowledge.

Acknowledgments

We would like to convey our deepest thanks and acknowledgement school directors and homeroom teachers for guiding us during students' filling of questionnaire and during collecting data on Mathematics scores from school documents. We also are grateful to students who participated in this study for their provision of the necessary data in this study by filling questionnaire. Our thanks also go to our colleagues whose suggestions in the entire project have had meaningful contribution to the accomplishment of this research output.

Author contributions

Conceptualization: Woldeab Daniel Eka, Tiruwork Tamiru Tola, Reda Darge Negasi.

Data curation: Woldeab Daniel Eka, Tiruwork Tamiru Tola, Reda Darge Negasi.

Formal analysis: Woldeab Daniel Eka, Tiruwork Tamiru Tola.

Funding acquisition: Tiruwork Tamiru Tola.

Investigation: Woldeab Daniel Eka, Reda Darge Negasi.

Methodology: Woldeab Daniel Eka, Tiruwork Tamiru Tola, Reda Darge Negasi.

Project administration: Woldeab Daniel Eka, Reda Darge Negasi.

Resources: Woldeab Daniel Eka.

Software: Woldeab Daniel Eka, Reda Darge Negasi.

Supervision: Tiruwork Tamiru Tola, Reda Darge Negasi.

Validation: Woldeab Daniel Eka, Reda Darge Negasi.

Visualization: Woldeab Daniel Eka, Tiruwork Tamiru Tola, Reda Darge Negasi.

Writing – original draft: Woldeab Daniel Eka.

Writing – review & editing: Woldeab Daniel Eka, Tiruwork Tamiru Tola, Reda Darge Negasi.

References

1. Ministry of Education (MoE) Federal Democratic Republic of Ethiopia. Curriculum Framework for Ethiopian Education (KG-Grade 12). Addis Ababa, Ethiopia: Ministry of Education (MoE) Federal Democratic Republic of Ethiopia. 2009.
2. Sutton J, Krueger A. What we know about mathematics teaching and learning. Washington DC, USA: Office of Midcontinent Research for Education and Learning. 2002.
3. Li Y, Schoenfeld AH. Problematizing teaching and learning mathematics as "given" in STEM education. IJ STEM Ed. 2019;6(1). https://doi.org/10.1186/s40594-019-0197-9
4. Kubiszyn T, Borich G. Educational testing and measurement: Classroom Application and Practice. Jefferson City, USA: John Wiley & Sons, Inc. 2013.
5. Zakaria E, Addenan N, Maat S, Nordin N. Teaching mathematics: understanding of concepts and the use of high-order cognitive strategie among secondary school teachers. In: International Conference on Mathematics, Science and Education, 2016.
6. Sbhatu D, Weldeana H. The effects of small-group cooperative learning strategy on college students' performance of basic mathematics. The Ethiopian Journal of Education. 2011;31(1):65–93.
7. Gersten R, Chard DJ, Jayanthi M, Baker SK, Morphy P, Flojo J. Mathematics Instruction for Students With Learning Disabilities: A Meta-Analysis of Instructional Components. Review of Educational Research. 2009;79(3):1202–42. https://doi.org/10.3102/0034654309334431
8. Appiah JB, Korkor S, Arthur YD, Obeng BA. Mathematics achievement in high schools, the role of the teacher-student relationship, students' self-efficacy, and students' perception of mathematics. INT ELECT J MATH ED. 2022;17(3):em0688. https://doi.org/10.29333/iejme/12056
9. Walde GS. The actual status of general secondary school mathematics teaching and learning: The case of Ethiopia. J Phys: Conf Ser. 2019;1176:042097. https://doi.org/10.1088/1742-6596/1176/4/042097
10. Lin S-W, Tai W-C. Latent Class Analysis of Students' Mathematics Learning Strategies and the Relationship between Learning Strategy and Mathematical Literacy. ujer. 2015;3(6):390–5. https://doi.org/10.13189/ujer.2015.030606
11. Nuutila K, Tapola A, Tuominen H, Molnár G, Niemivirta M. Mutual relationships between the levels of and changes in interest, self-efficacy, and perceived difficulty during task engagement. Learning and Individual Differences. 2021;92:102090. https://doi.org/10.1016/j.lindif.2021.102090
12. Schunk D. Learning theories: An educational perspective. 6 ed. Boston: Pearson Education, Inc. 2012.
13. Tella A. Teacher variables as predictors of academic achievement of primary school pupils' mathematics. International Electronic Journal of Elementary Education. 2008;1(1):16–33.
14. Ministry of Education headquarters. Grade 12 Exams: Over 36, 400 Students Score 50% and Above. 2024. https://ethiopianmonitor.com/2024/09/09/grade-12-exams-over-36-400-students-score-50-and-above/
15. Sime G. Problems influencing academic performance of students in government secondary schools of Oromia Special Zone. Ethiopia: Addis Ababa University. 2018. https://etd.aau.edu.et/server/api/core/bitstreams/ba16c9f1-c6e0-454b-9fb4-f0919a35ed5e/content
16. Tadesse A, Kenea A. The practice of learning post-1994 instructional reforms in Ethiopia: Looking a case primary school through the lens of organizational learning theory. Cogent Education. 2022;9(1). https://doi.org/10.1080/2331186x.2022.2074343
17. Tiruneh D, Hoddinott J, Rolleston C, Sabates R, Woldehanna T. Understanding achievement in numeracy among primary school children in Ethiopia: evidence from rise Ethiopia study. RISE Working Paper Series. 2021;21(071). https://doi.org/10.35489/BSG-RISE-WP_2021/071
18. Magen Nagar N. The Effects of Learning Strategies on Mathematical Literacy: A Comparison between Lower and Higher Achieving Countries. IJRES. 2016;2(2):306. https://doi.org/10.21890/ijres.77083

19. Obergriesser S, Stoeger H. Students' emotions of enjoyment and boredom and their use of cognitive learning strategies – How do they affect one another?. Learning and Instruction. 2020;66:101285. https://doi.org/10.1016/j.learninstruc.2019.101285
20. Rittle-Johnson B, Star JR, Durkin K. Developing procedural flexibility: are novices prepared to learn from comparing procedures?. Br J Educ Psychol. 2012;82(Pt 3):436–55. https://doi.org/10.1111/j.2044-8279.2011.02037.x PMID: 22881048
21. Weinstein E, Mayer E. The Teaching of Learning Strategies. Innovation Abstracts. 1983;5(32).
22. Kumar S, Jagacinski CM. Confronting task difficulty in ego involvement: Change in performance goals. Journal of Educational Psychology. 2011;103(3):664–82. https://doi.org/10.1037/a0023336
23. Barch DM, Braver TS, Nystrom LE, Forman SD, Noll DC, Cohen JD. Dissociating working memory from task difficulty in human prefrontal cortex. Neuropsychologia. 1997;35(10):1373–80. https://doi.org/10.1016/s0028-3932(97)00072-9 PMID: 9347483
24. Landicho R. Factors affecting performance in general mathematics of grade eleven students in Talumpok integrated school: Basis for intervention activities. International Journal of Innovative Science and Research Technology. 2021;6(1).
25. Bandura A. Social foundations of thought and action: A social cognitive theory. Englewood Cliffs: Prentice-Hall. 1986.
26. Lo Schiavo M, Prinari B, Saito I, Shoji K, Benight CC. A dynamical systems approach to triadic reciprocal determinism of social cognitive theory. Mathematics and Computers in Simulation. 2019;159:18–38. https://doi.org/10.1016/j.matcom.2018.10.006
27. Paderan M. Math strategies used by secondary public school teachers. International Journal of Open-Access, Interdisciplinary & New Educational Discoveries. 2023;2(3):13–23.
28. Tezer M, Gülyaz Cumhur M, İldırımlı A. Examination of Mathematics Study Strategies of Secondary School Students From the Perspective of Multiple Variables. IJCRSEE. 2020;8(3):83–92. https://doi.org/10.23947/2334-8496-2020-8-3-83-92
29. Qu Y, Wang C, Zhong L. The Research and Discussion of Web-Based Adaptive Learning Model and Strategy. Lecture Notes in Computer Science. Springer Berlin Heidelberg. 2009. 412–20. https://doi.org/10.1007/978-3-642-03697-2_38
30. Buhagiar D. Views of Mathematics. Jesuit in Malta. 2013.
31. Rodríguez S, Regueiro B, Piñeiro I, Valle A, Sánchez B, Vieites T, et al. Success in Mathematics and Academic Wellbeing in Primary-School Students. Sustainability. 2020;12(9):3796. https://doi.org/10.3390/su12093796
32. Almutlaq SA. Buffering the Effects of Students' Perceived Task Difficulty on Cognitive Engagement in EFL Writing Classrooms: The Role of Foreign Language Enjoyment and Growth Mindset. JLTR. 2024;15(5):1478–88. https://doi.org/10.17507/jltr.1505.09
33. Mutodi P, Ngirande H. The Influence of Students` Perceptions on Mathematics Performance. A Case of a Selected High School in South Africa. MJSS. 2014. https://doi.org/10.5901/mjss.2014.v5n3p431
34. Saccoletto M, Spagnolo C. Students' perceived difficulty of mathematical tasks: an investigation on influencing factors. didmat. 2022;44(1). https://doi.org/10.14708/dm.v44i1.7181
35. Pohl AJ. Strategies and Interventions for Promoting Cognitive Engagement. Student Engagement. Springer International Publishing. 2020. 253–80. https://doi.org/10.1007/978-3-030-37285-9_14
36. Schunk D, Pintrich P, Meece J. Motivation in education: Theory, research, and applications. 3 ed. Upper Saddle River, NJ: Pearson Education. 2008.
37. Wigfield A, Cambria J, Eccles J. Motivation in education. Ryan RM. Oxford University Press. 2012.
38. Lee J-S. The Relationship Between Student Engagement and Academic Performance: Is It a Myth or Reality?. The Journal of Educational Research. 2013;107(3):177–85. https://doi.org/10.1080/00220671.2013.807491
39. Pintrich PR. A Motivational Science Perspective on the Role of Student Motivation in Learning and Teaching Contexts. Journal of Educational Psychology. 2003;95(4):667–86. https://doi.org/10.1037/0022-0663.95.4.667
40. Ryan RM, Deci EL. Brick by Brick: The Origins, Development, and Future of Self-Determination Theory. Advances in Motivation Science. Elsevier. 2019. 111–56. https://doi.org/10.1016/bs.adms.2019.01.001
41. Mertens D. Research and evaluation in education and psychology: integrating diversity with quantitative, qualitative and mixed methods. 3 ed. Thousand Oaks, California: SAGE Publications, Inc. 2010.
42. Yamane T. Statistics: An Introductory Analysis. 2 ed. New York: Harper and Row. 1967.
43. Kaldo I, Õun K. Gender differences favouring females in learning strategies in mathematics. PEC. 2020;78(4):595–611. https://doi.org/10.33225/pec/20.78.595
44. Kyndt E, Dochy F, Struyven K, Cascallar E. The perception of workload and task complexity and its influence on students' approaches to learning: a study in higher education. Eur J Psychol Educ. 2010;26(3):393–415. https://doi.org/10.1007/s10212-010-0053-2
45. Collier E. Applied Structural Equation Modeling Using AMOS. New York and London: Taylor and Francis Group. 2020.
46. Ho R. Handbook of univariate and multivariate data analysis and interpretation with SPSS. Boca Raton, London, New York: Taylor & Francis Group. 2006.
47. Pallant J. SPSS: Survival Manual A Step by Step Guide to Data Analysis Using SPSS. 4 ed. New York, USA: McGraw-Hill Education. 2010.
48. Zeller RA. Measurement Error, Issues and Solutions. Encyclopedia of Social Measurement. Elsevier. 2005. 665–76. https://doi.org/10.1016/b0-12-369398-5/00109-2
49. Leech LN, Barrett CK, Morgan AG. SPSS for intermediate statistics use and interpretation. 2 ed. Mahwah, New Jersey, London: Lawrence Erlbaum Associates, Inc. 2005.

50. Tabachnick GB, Fidell S. Using multivariate and univariate statistics. 6 ed. Boston, New York, London: Pearson Education, Inc. 2013.
51. Mulisa F, Kassahun S. The roles of a peer-led collaborative learning approach in Ethiopian secondary schools. Bahir Dar Journal of Education. 2018;18(2).
52. Chun J, Cennamo KA. A theoretical model of peer learning incorporating scaffolding strategies. International Journal of Teaching and Learning in Higher Education. 2022;22(3):385–97.
53. Azevedo R. Computer Environments as Metacognitive Tools for Enhancing Learning. Educational Psychologist. 2005;40(4):193–7. https://doi.org/10.1207/s15326985ep4004_1
54. Belo NAH, van Driel JH, van Veen K, Verloop N. Beyond the dichotomy of teacher- versus student-focused education: A survey study on physics teachers' beliefs about the goals and pedagogy of physics education. Teaching and Teacher Education. 2014;39:89–101. https://doi.org/10.1016/j.tate.2013.12.008
55. Zengin Y, Tatar E. Integrating dynamic mathematics software into cooperative learning environments in mathematics. Educational Technology and Society. 2017;20(2).
56. Talib A, Kailani IB. Problem Based Learning in Cooperative Situation (PBLCS) and Its Impact on Development of Personal Intelligence. International Journal of Evaluation and Research in Education (IJERE). 2014;3(4). https://doi.org/10.11591/ijere.v3i4.6969
57. McInnis C. New realities of the student experience: how should universities respond?. In: Limerick, Ireland. 2003.
58. Almoslamani Y. The impact of learning strategies on the academic achievement of university students in Saudi Arabia. Learning and Teaching in Higher Education. Gulf Perspectives. 2022;18(1):4–18. https://doi.org/10.1108/LTHE-08-2020-0025
59. Chiu MM, Chow BW-Y, Mcbride-Chang C. Universals and specifics in learning strategies: Explaining adolescent mathematics, science, and reading achievement across 34 countries. Learning and Individual Differences. 2007;17(4):344–65. https://doi.org/10.1016/j.lindif.2007.03.007
60. Saraswathy R. Learning strategies in mathematics among secondary school boys and girls in relation to their academic achievement. International Journal of Science & Engineering Development Research. 2019;4(7):356–9.
61. Bjork E, Bjork R. Making things hard on yourself, but in a good way: Creating desirable difficulties to enhance learning. Psychology and the real world. 2011. 55–64.
62. Türker Biber B. Self-Regulated Learning Strategies Used by Students to Prepare Mathematics Exams. IJPE. 2022;18(4):223–38. https://doi.org/10.29329/ijpe.2022.459.16
63. Greene BA, Miller RB, Crowson HM, Duke BL, Akey KL. Predicting high school students' cognitive engagement and achievement: Contributions of classroom perceptions and motivation. Contemporary Educational Psychology. 2004;29(4):462–82. https://doi.org/10.1016/j.cedpsych.2004.01.006
64. Lannie AL, Martens BK. Effects of task difficulty and type of contingency on students' allocation of responding to math worksheets. J Appl Behav Anal. 2004;37(1):53–65. https://doi.org/10.1901/jaba.2004.37-53 PMID: 15154215
65. Li W, Lee A, Solmon M. The role of perceptions of task difficulty in relation to self-perceptions of ability, intrinsic value, attainment value, and performance. European Physical Education Review. 2007;13(3):301–18. https://doi.org/10.1177/1356336x07081797
66. Fulmer SM, Tulis M. Changes in interest and affect during a difficult reading task: Relationships with perceived difficulty and reading fluency. Learning and Instruction. 2013;27:11–20. https://doi.org/10.1016/j.learninstruc.2013.02.001
67. Fulmer SM, Frijters JC. Motivation During an Excessively Challenging Reading Task: The Buffering Role of Relative Topic Interest. The Journal of Experimental Education. 2011;79(2):185–208. https://doi.org/10.1080/00220973.2010.481503
68. Capa RL, Audiffren M, Ragot S. The effects of achievement motivation, task difficulty, and goal difficulty on physiological, behavioral, and subjective effort. Psychophysiology. 2008;45(5):859–68. https://doi.org/10.1111/j.1469-8986.2008.00675.x PMID: 18627535
69. Hattie J. Visible learning: A synthesis of over 800 meta-analyses relating to achievement. New York: Taylor & Francis. 2009.
70. Lynch R, Patten JV, Hennessy J. The impact of task difficulty and performance scores on student engagement and progression. Educational Research. 2013;55(3):291–303. https://doi.org/10.1080/00131881.2013.825165
71. Kamata A, Nese JFT, Patarapichayatham C, Lai C-F. Modeling Nonlinear Growth With Three Data Points. Assessment for Effective Intervention. 2012;38(2):105–16. https://doi.org/10.1177/1534508412457872
72. Boaler J, Dieckmann JA, Pérez-Núñez G, Sun KL, Williams C. Changing Students Minds and Achievement in Mathematics: The Impact of a Free Online Student Course. Front Educ. 2018;3. https://doi.org/10.3389/feduc.2018.00026
73. Bekdemir M. The pre-service teachers' mathematics anxiety related to depth of negative experiences in mathematics classroom while they were students. Educ Stud Math. 2010;75(3):311–28. https://doi.org/10.1007/s10649-010-9260-7
74. Brunstein JC, Schmitt CH. Assessing individual differences in achievement motivation with the Implicit Association Test. Journal of Research in Personality. 2004;38(6):536–55. https://doi.org/10.1016/j.jrp.2004.01.003
75. van Steensel R, Oostdam R, van Gelderen A. Affirming and undermining motivations for reading and associations with reading comprehension, age and gender. Journal Research in Reading. 2019;42(3–4):504–22. https://doi.org/10.1111/1467-9817.12281
76. Gee JP. A Sociocultural Perspective on Opportunity to Learn. Assessment, Equity, and Opportunity to Learn. Cambridge University Press. 2008. 76–108. https://doi.org/10.1017/cbo9780511802157.006
77. Mohammed Shuker F, Hashim Sadik H. A critical review on rural youth unemployment in Ethiopia. International Journal of Adolescence and Youth. 2024;29(1). https://doi.org/10.1080/02673843.2024.2322564
